# Differences in eating habits, physical activity, and sleep patterns among high school students and their parents before and during COVID-19 pandemic

**DOI:** 10.3389/fpubh.2024.1408145

**Published:** 2024-07-19

**Authors:** Shoug Alashmali

**Affiliations:** Department of Clinical Nutrition, Faculty of Applied Medical Sciences, King Abdulaziz University, Jeddah, Saudi Arabia

**Keywords:** COVID-19, eating habits, physical activity, sleep, high school students, parents, Saudi Arabia

## Abstract

**Introduction:**

The recent COVID-19 pandemic has profoundly disrupted the eating and lifestyle habits among populations, especially among younger populations who are more prone to unhealthy dietary behaviours. However, current knowledge related to eating and lifestyle patterns, especially physical activity and sleep among different generations is limited. Our study sought to understand the eating habits, physical activity, and sleep behaviours among high school students compared to their parents in Saudi Arabia, before and during the COVID-19 pandemic.

**Methods:**

This study was conducted in public high schools in Jeddah, Saudi Arabia (*n* = 8 schools), spanning from September 2021 to April 2022. Data were collected using an online questionnaire, distributed to all students and their parents (*n* = 516) in these schools.

**Results:**

During the pandemic, parents (46.4%) showed a heightened interest in health and nutrition compared to students (32.7%, *p* = 0.001). Food preferences changed for 58.1% of participants; 70.0% of parents and 36.7% of students opted for homemade food (*p* < 0.001). Unhealthy behaviours like eating out were more common in students. Weight varied by 74.0% during COVID-19, with more parents (41.4%) gaining weight than students (31.6%, *p* = 0.018). Physical activity dropped more for parents (42.1%) than students (23.9%), though 30.7 and 31.6% reported increases, respectively (*p* < 0.001). Regardless of the pandemic, students were more likely to sleep later compared to parents (58.0% vs. 41.4%; *p* < 0.001), while parents were more prone to waking up earlier compared to students (81.4% vs. 67.3%, *p* = 0.002).

**Discussion:**

The findings underscore the varied pandemic impact on eating habits and physical activity between students and parents. Tailored interventions are vital for promoting healthier choices during health crises.

## 1 Introduction

Eating habits regroup “conscious, collective, and repetitive behaviours, which lead people to select, consume, and use certain foods or diets, in response to social and cultural influences” (1). Eating habits are shaped by several individual, societal, and environmental influences, such as food taste, and culinary culture, influence of peers and society, and availability of cooking resources and food prices (2). Given the role of a healthy diet in disease prevention and health promotion, it is essential to assess dietary patterns across populations (3). The World Health Organization advises a diet rich in nutrient-dense food, balanced energy intake and expenditure, and minimal consumption of saturated fats, sugars, and salts (4). Fast food, a primary contributor to poor diet, is linked to various cardiometabolic disorders due to its high-calorie, sugar, salt, and fat content, alongside carbonated drinks (5).

Adolescent fast-food consumption, particularly among high school students, is linked to early onset obesity and its long-term risks (6). In countries like the United States, over a third of this demographic consumed fast food daily between 2015 and 2018. However, this trend extends beyond developed nations (7). According to a recent Global School-Based Student Health Survey across 54 low-to-middle-income countries, over half of the adolescents consumed fast food weekly, with 10.3% consuming it four to seven times a week, the highest rates noted in Southeast Asia (7). In Saudi Arabia too, adolescents show a predilection for fast food and carbonated drinks over fruits and vegetables (8, 9).

Decreased physical activity, alongside fast-food consumption, is another major unhealthy lifestyle trend among adolescents. Physical activity levels among Saudi Arabian adolescents show wide disparities, ranging from 4 to 44.5% (8). This indicates a marked inadequacy in physical activity levels within this demographic, underscoring an area of concern in adolescent health behaviour. Further, sleep deprivation is increasingly prevalent among adolescents, including those in Saudi Arabia (8, 10).

Besides these trends, the recent COVID-19 pandemic has raised additional concerns regarding disruptions to eating habits and lifestyle patterns such as physical activity and sleep among populations. While the pandemic has encouraged some individuals to adopt healthier lifestyles, it has conversely led others towards less healthy behaviours. An Italian study involving individuals aged 12 and above showed that the COVID-19 pandemic and subsequent lockdown led to weight gain among nearly half of the respondents. On the other hand, nearly 40% have increased their physical activity, 15% shifted to purchasing organic produce, and a minority (3.3%) of smokers decided to quit. Additionally, adherence to the Mediterranean diet was higher among the 18–30 age group (11). In Spain, approximately 40% gained weight and 31% lost weight, while nearly 40% reported poorer sleep quality. Both weight gain and poorer sleep were correlated with age and BMI. Physical activity ceased in 44.7% of participants, varying by sex, age, BMI, and sleep quality. Based on an emotional-eater questionnaire, 21.8 and 11% were classified as emotional eaters or very emotional eaters, respectively (12). In the United Arab Emirates (UAE), the COVID-19 period was marked by weight gain among 31% of the individuals, associated with high rates of physical inactivity (38.5%), along with a shift from Mediterranean diet towards unhealthy patterns. Additionally, emotional and physical exhaustion and irritability were significantly higher during the pandemic compared to before, and sleep disturbances were reported in 60.8% of participants (13). In Kuwait, among 415 participants aged 18–73, the practice of skipping breakfast remained consistent, while late-night snack or meal consumption significantly increased during the pandemic. Interestingly, fast-food consumption showed a drastic decrease, with 82% of participants reporting no consumption during the COVID-19 period. The majority of participants also reported having freshly made meals as their main dish. There was also a significant increase in Americano coffee and fresh juice consumption, and a decrease in fish and seafood intake. However, there was a significant reduction in physical activity, an increase in screen time and sedentary behaviours, and altered sleep patterns with increased day-time sleep and decreased night-time sleep (14).

These observations stress the relevance of assessing the changes in eating habits and lifestyle parameters after COVID-19, especially among younger populations who are more prone to unhealthy dietary behaviours (15). Therefore, the present study aimed to explore before and during COVID-19 changes in eating habits and lifestyle behaviours including physical activity and sleep among Saudi high school students, by reference to older populations represented by their respective parents. Understanding these pandemic-induced eating and lifestyle shifts is crucial for informing public health strategies and educational programs aimed at promoting healthier choices, particularly among young and at-risk populations, who seem to be more susceptible to such detrimental changes.

## 2 Materials and methods

### 2.1 Study design

We conducted a cross-sectional pilot study in public high schools in Jeddah, Saudi Arabia, spanning from September 2021 to April 2022. It was conducted according to the guidelines proposed in the Declaration of Helsinki and was reviewed and approved by the Research Ethics Committee of the Faculty of Applied Medical Sciences at King Abdulaziz University on November 2, 2021, under the reference number FAMS-EC2021-15. The Planning and Information Department of the Ministry of Education in Jeddah City granted us permission to distribute the survey electronically to both boys’ and girls’ high schools.

### 2.2 Participants and sampling

The study included students (both boys and girls) aged 15–18 years, enrolled in grades 10, 11, and 12, as well as their parents residing in Jeddah city. Students outside this age range and those with any chronic diseases were excluded from the study. The initial sample consisted of 528 students, of whom 376 (71%) met the inclusion criteria.

**Figure 1:**
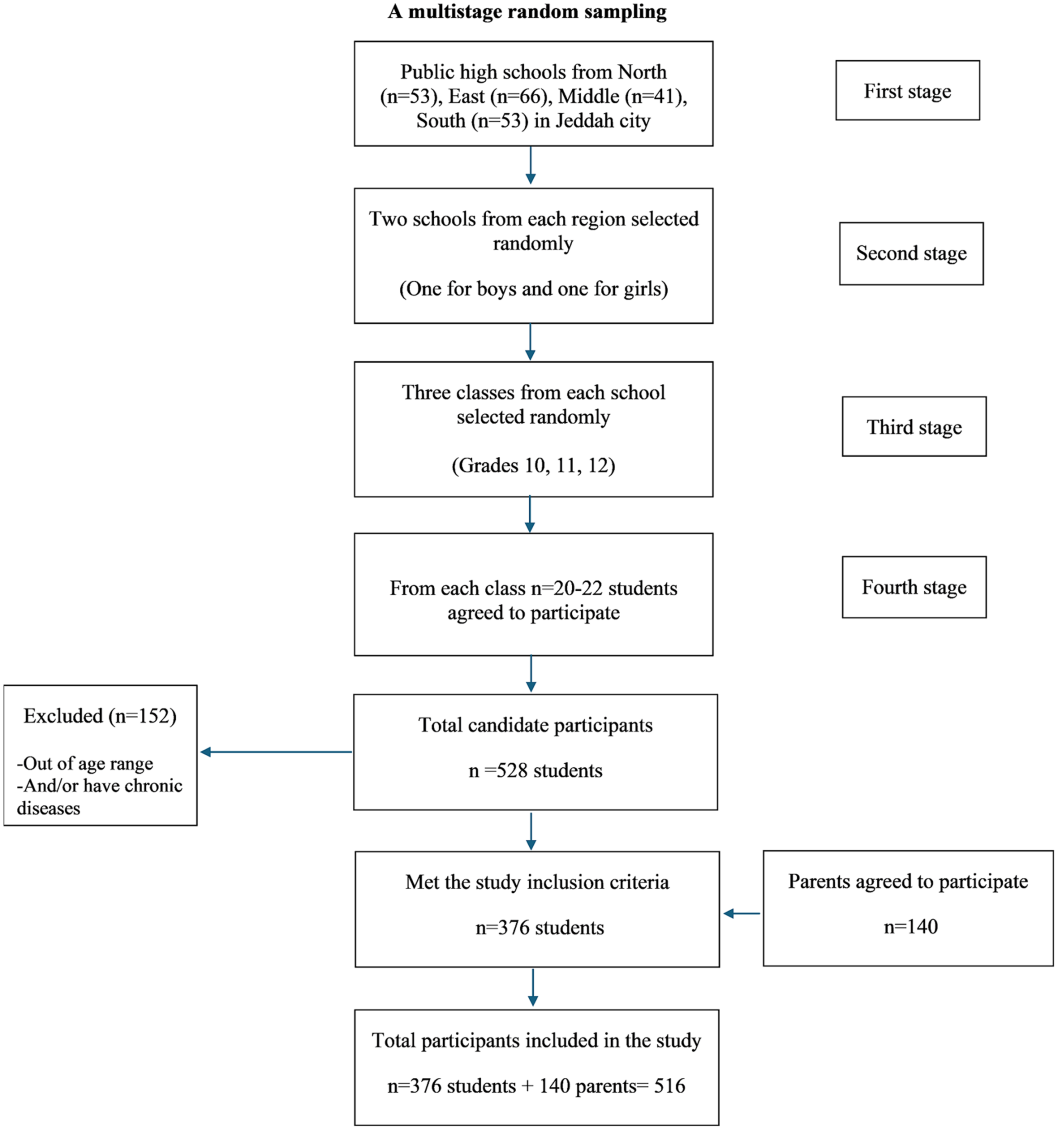
Flowchart for eligible sample.

We employed a multistage random sampling technique to select student participants from schools in Jeddah city, which is geographically divided into four regions according to the local school supervisory centers (North, East, Middle, and South). The first stage was to proportionally allocate the number of schools across these four regions (*n* = 213) (Figure 1). In the second stage, we randomly selected two schools from each region: one for boys and one for girls. From each school, three classes were chosen at random (one class from each grade: 10th, 11th, and 12th). Each class was treated as a cluster, and all students in that class, along with their parents, were invited to participate in the study. On average, there were 20–22 students per class in each school. The study protocol and procedures were explained at the beginning of the survey and written informed consent was obtained from all participants prior to their participation.

In total, 376 students and 140 parents participated in the study. We surveyed eight schools, encompassing 24 classes from grades 10th, 11th, and 12th. Of the student sample, 147 were from boys’ schools, while 229 were from girls’ schools.

An online version of the questionnaire was tested on a smaller sample of 94 participants (58 students and 36 parents), who were not included in the parent study. Minor amendments were made on the questionnaire to enhance clarity.

### 2.3 Sample size

We calculated the required sample size using the Epi Info sample size calculator, considering the total number of high school students in Jeddah to be 153,641 students (General Directorate of Education in Jeddah, Saudi Arabia). We estimated a dropout rate of 20% and applied a confidence level of 95%, a margin error of 5%, and a design effect of 1:1. Therefore, we determined the total sample size to be 245 students.

We exceeded the student target by recruiting 376 participants; however, we faced challenges in recruiting parents, resulting in a smaller sample size of only 140 participants. The limited access to parents compared to students contributed to this discrepancy. Parental participation was encouraged through indirect contact, involving communication via schools’ management. In contrast, student recruitment involved direct engagement. This difference in recruitment methods made it more difficult to recruit parents, which is the primary reason for the disparity in sample sizes between students and parents.

### 2.4 Data collection

An online questionnaire was designed to evaluate eating, physical activity, and sleep patterns both before and during the COVID-19 pandemic among high school students and their parents. The questionnaire that has been used in this study was adapted from the works of Aljaaly (16) and Bakhsh et al. (17) in Arabic Language. After that an expert panel, consisting of eight health professionals with medical and clinical nutrition backgrounds reviewed, revised, and approved the Arabic version of the questionnaire, using the guidelines provided by Tsang et al. (18). Prior to administration, the questionnaire was pilot tested among a sample of 92 respondents, who were not included in the study. The pilot sample provided their feedback regarding the clarity of the items, thereby enabling editing of the final version of the questionnaire.

The questionnaire included four sections. Section one encompassed ten questions relating to socio-demographic and health-related characteristics such as age, gender, nationality, place of residence, marital status, education level, work status, history/type of chronic diseases, and COVID-19. Section two evaluated eating habits and sleep patterns before COVID-19. We inquired about participants’ regular meals, snacking habits, home-cooked food consumption, eating out habits, beverage choices, frequency of water intake, and sleep/wake patterns. Section three explored the impact of COVID-19 and lockdown on eating habits and physical activity. We evaluated how the interest towards health and nutrition changed during COVID-19 (not affected, decreased, increased), as well as the shifts in food preference (lesser or more homemade, etc.), number of meals and snacks, and exercise, and the patterns of weight changes (no change vs. gain vs. loss). Section four investigated the knowledge about nutrition and health between students and parents before and during the pandemic. We collected information related to reading or following about weight, diet, and nutrition, source of this information, and whether the interest to know about health and nutrition affected by COVID-19.

All the four sections of the questionnaire including demographic and health status, eating habits and sleep patterns before COVID-19, eating habits and physical activity during COVID-19, and the knowledge about nutrition and health were mandatory to be answered by the participants who agreed, based on the consent form, to participate in the study.

### 2.5 Statistical analysis

We used IBM SPSS Statistics, version 21.0 for Windows, for data analysis. Descriptive statistics were used to present the answers to the different questionnaire sections. The differences in eating habits and lifestyle patterns including physical activity and sleep patterns between the two generations (students versus parents), as well as the impact of COVID-19 of these patterns, were analyzed using Chi-square and Fischer’s exact tests, as applicable. A *p*-value <0.05 was considered statistically significant.

## 3 Results

### 3.1 Characteristics of the study participants

This survey was completed by 516 participants, including 376 high school students (147 males and 229 females) and 140 parents (47 males and 93 females). The family size was 6 or more in 71.2% of the participants. Majority of the parents were aged 41–50 years (54.3%), followed by 22–40 years (27.1%), and 71.4% of them had a bachelor’s degree. Students were aged 15–18 years in 91.2% of the cases. Chronic diseases were more frequent among parents (22.9%) than students (9.6%), *p* < 0.001. COVID-19 infection was reported among 26.6% of the students and 29.3% of the parents (Table 1).

**Table 1:** Characteristics of the study participants (*N* = 516).

Item	Level	Total (*N* = 516)	Students (*N* = 376)	Parents (*N* = 140)	*p*-value
Gender	Male	194 (37.6)	147 (39.1)	47 (33.6)	
	Female	322 (62.4)	229 (60.9)	93 (66.4)	0.250
Age group	15–18	376 (72.9)	376 (100)	0 (0)	
	19–21	0 (0)	0 (0)	0 (0)	
	22–40	41 (7.9)	0 (0)	41 (29.3)	
	41–50	78 (15.1)	0 (0)	78 (55.7)	
	51–60	19 (3.7)	0 (0)	19 (13.6)	
	>61	2 (0.4)	0 (0.0)	2 (1.4)	<0.001*
Parents marital status	No data	368 (71.3)	363 (96.5)	5 (3.6)	
	Died	2 (0.4)	1 (0.3)	1 (0.7)	
	Divorced	10 (1.9)	1 (0.3)	9 (6.4)	
	Married	129 (25.0)	10 (2.7)	119 (85.0)	
	Widowed	7 (1.4)	1 (0.3)	6 (4.3)	<0.001*
Family members number	1–5	148 (28.7)	95 (25.3)	53 (37.9)	
	6–9	332 (45.7)	250 (66.6)	82 (62.1)	
	10+	36 (6.9)	31 (8.4)	5 (3.5)	0.239
Parents’ educational level	No data	368 (71.3)	363 (95.5)	5 (3.5)	
	Bachelor	104 (20.2)	4 (1.1)	100 (71.4)	
	Elementary	6 (1.2)	1 (0.3)	5 (3.6)	
	Intermediate	7 (1.4)	2 (0.5)	5 (3.6)	
	Secondary	31 (6.0)	6 (1.3)	25 (17.9)	<0.001*
Employment	No data	368 (71.3)	363 (96.5)	5 (3.6)	
	No	51 (9.9)	8 (2.1)	43 (30.7)	
	Yes	97 (18.8)	5 (1.3)	92 (65.7)	<0.001*
Job type	No data	420 (81.4)	372 (98.9)	48 (34.5)		Education	48 (9.3)	2 (0.5)	46 (32.9)		Health	6 (1.2)	0 (0.0)	6 (4.3)		Management private	18 (3.5)	2 (0.5)	16 (11.4)		Management public	20 (3.9)	0 (0.0)	20 (14.3)		Private	3 (0.6)	0 (0.0)	3 (2.1)		Retired military	1 (0.2)	0 (0.0)	1 (0.7)	<0.001*
Chronic diseases	No data	2 (0.4)	2 (0.5)	0 (0.0)		No	446 (86.4)	338 (89.9)	108 (77.1)		Yes	68 (13.2)	36 (9.6)	32 (22.9)	<0.001*
Type of chronic disease	No data	449 (87.0)	341 (90.7)	108 (77.1)	
	Blood diseases (Thalassemia)	3 (0.6)	3 (0.8)	0 (0.0)	
	Respiratory diseases (bronchitis, COPD, sinusitis)	14 (2.7)	10 (2.7)	4 (2.8)	
	Cancer	1 (0.2)	1 (0.3)	0 (0.0)	
	Depression and anxiety	4 (0.8)	3 (2.8)	1 (0.7)	
	Diabetes	15 (2.9)	5 (1.3)	10 (7.1)	
	Food allergy	6 (1.2)	4 (1.1)	2 (1.4)	
	GIT disorders	6 (1.2)	4 (1.1)	2 (1.4)	
	Heart diseases (Hypertension, hyperlipidemia)	7 (1.4)	0 (0.0)	7 (5.0)	
	AIDS	1 (0.2)	0 (0.0)	1 (0.7)		
	Others (SLE, allergy, Addison disease, epilepsy, hypothyroidism, obesity)	8 (2.2)	4 (1.2)	4 (2.8)	<0.001*
Infected by COVID-19	No data	163 (31.6)	104 (27.7)	59 (42.1)	
	No	212 (41.1)	172 (45.7)	40 (28.6)	
	Yes	141 (27.3)	100 (26.6)	41 (29.3)	<0.001*

*p value significant < 0.05, and highlighted in bold. Chi square or Fischer’s exact test were used to identify significance. AIDS, acquired immunodeficiency syndrome; COPD, chronic obstructive pulmonary disease; GIT, gastrointestinal tract; SLE, systemic lupus erythematosus.

### 3.2 Eating habits and sleep patterns before COVID-19 pandemic

A significant proportion of participants, particularly students (64.6%), did not adhere to eating three main meals daily compared to parents (42.1%), and the difference was statistically significant (*p* < 0.001). Most meals were prepared by mothers for students (78.5%) and wives for parents (55.7%), with a significant difference observed (*p* < 0.001). Participants predominantly favored home-made food (68.8%), more among parents than students, respectively (92.1% vs. 60.1%, *p* < 0.001). Notably, there was a higher preference for eating out (86.2% vs. 76.4%; *p* = 0.009) and consuming more than one take-out meal (45.2% vs. 27.9%; *p* = 0.001) among students versus parents, respectively. Fast food was preferred over casual dining, especially among students (*p* < 0.001). Water was the most consumed beverage in both groups, while significant differences were observed in other types of beverages (*p* < 0.001). Distinct sleep patterns were evident between students and parents, as students were more likely to sleep later compared to parents (58.0% vs. 41.4%; *p* < 0.001), while parents were more prone to waking up earlier compared to students (81.4% vs. 67.3%, *p* = 0.002) respectively (Table 2).

**Table 2:** Eating habits and sleep patterns before COVID-19 in students and parents.

Eating habit	Level	Total (*N* = 516)	Students (*N* = 376)	Parents (*N* = 140)	*P*-value
		*N* (%)	*N* (%)	*N* (%)	
Three main meals	No	302 (58.5)	243 (64.6)	59 (42.1)	
	Yes	214 (41.5)	133 (35.4)	81 (57.9)	<0.001*
Snacks	No	121 (23.4)	85 (22.6)	36 (25.7)	
	Yes	395 (76.6)	291 (77.4)	104 (74.3)	0.606
Meal prepared by	Mother	328 (63.6)	295 (78.5)	33(23.6)	
	Self	100 (19.4)	71 (18.9)	29 (20.7)	
	Wife	88 (17.1)	10 (2.7)	78 (55.7)	<0.001*
Favorite food	Home-made	355 (68.8)	226 (60.1)	129 (92.1)	
	Ready meals	161 (31.2)	150 (39.9)	11 (7.9)	<0.001*
Eating out	No	85 (16.5)	52 (13.8)	33 (23.6)	
	Yes	431 (83.5)	324 (86.2)	107 (76.4)	0.009*
Number of take-out meals	None	86 (16.7)	53 (14.1)	33 (23.6)	
	1	221 (42.8)	153 (40.7)	68 (48.6)	
	2–3	161 (31.2)	128 (34.0)	33 (23.6)	0.001*
	>3	48 (9.3)	42 (11.2)	6 (4.3)	
Type of restaurants	Casual	164 (31.8)	104 (27.7)	60 (42.9)	
	Fast food	267 (51.7)	220 (58.5)	47 (33.6)	<0.001*
Type of beverages	Fresh fruits juice	43 (8.3)	32 (8.5)	11 (7.9)	
	Hot drinks	80 (15.5)	41 (10.9)	39 (27.9)	
	Sugar-free CD	26 (5.0)	19 (5.1)	7 (5.0)	
	Sugar-sweetened CD	89 (17.2)	79 (21.0)	10 (7.1)	
	Sugar-sweetened drinks	23 (4.5)	23 (6.1)	0 (0.0)	
	Water	255 (49.4)	182 (48.4)	73 (52.1)	<0.001*
Number of daily water cups (*n* = 515)	1	35 (6.8)	34 (9.1)	2 (1.4)	
	2–3	181 (35.1)	136 (36.2)	45 (32.1)	
	4–6	189 (36.6)	132 (35.1)	58 (41.4)	
	7 and more	109 (21.1)	74 (19.7)	35 (25.0)	0.036*
Sleeping early	No	276 (53.5)	218 (58.0)	58 (41.4)	
	Yes	240 (46.5)	158 (42.0)	82 (58.6)	<0.001*
Waking up early	No	149 (28.9)	123 (32.7)	26 (18.6)	0.002*
	Yes	367 (71.1)	253 (67.3)	114 (81.4)	

CD, Carbonated drinks.

*p value significant < 0.05, and highlighted in bold. Chi square or Fischer’s exact test were used to identify significance.

### 3.3 Impact of COVID-19 on eating habits and physical activity

COVID-19 had marked effects on the eating habits and physical activity parameters. Food preferences shifted among 58.1% of the participants, with 70.0% of parents and 36.7% of students consuming more homemade food (*p* < 0.001). Changes in meal frequency were notable, with a quarter (25.0%) of parents increasing their number of meals versus 18.1% among students (*p* = 0.035). Weight fluctuations were observed among 74.0% of the participants during COVID-19, more often consisting of weight gain. Intergroup comparison showed that 41.4% of parents and 31.6% of students gained weight, while 16.4 and 25.0% lost weight, respectively (*p* = 0.018). There were significant changes in exercise time as well, with a substantial decrease particularly noted among 42.1% of parents compared to 23.9% of students, while 30.7 and 31.6% reported increase during COVID-19, respectively (*p* < 0.001) (Table 3).

**Table 3:** Impact of COVID-19 on eating habits and physical activity in students and parents.

Effect of COVID-19	Level	Total (*N* = 516)	Students (*N* = 376)	Parents (*N* = 140)	*p*-value
Food preference differed during COVID-19	No	216 (41.9)	184 (48.9)	32 (22.9)	
	Yes	300 (58.1)	192 (51.1)	108 (77.1)	<0.001*
How different?	Lesser homemade food	4 (0.8)	3 (0.8)	1 (0.7)	0.881
	Lesser ready meals	1 (0.2)	1 (0.3)	0 (0.0)	0.322
	More homemade food	236 (45.7)	138 (36.7)	98 (70.0)	<0.001*
	More ready meals	59 (11.4)	50 (13.3)	9 (6.4)	0.921
Change in number of meals during COVID-19	Not affected	283 (54.8)	203 (54.0)	80 (57.1)	
	Decreased	130 (25.2)	105 (27.9)	25 (17.9)	
	Increased	103 (20.0)	68 (18.1)	35 (25.0)	0.035*
Change in number of snacks during COVID-19	Not affected	261 (50.6)	188 (50.0)	73 (52.1)	
	Decreased	103 (20.0)	80 (21.3)	23 (16.4)	
	Increased	152 (29.5)	108 (28.7)	44 (31.4)	0.461
Weight changes during COVID-19	Not changed	134 (26.0)	92 (24.5)	42 (30.0)	
	Changed *(how)*	382 (74.0)	284 (75.5)	98 (70.0)	0.018*
	Gained	177 (34.3)	119 (31.6)	58 (41.4)	
	Lost	117 (22.7)	94 (25.0)	23 (16.4)	
	Not sure	88 (17.1)	71 (18.9)	17 (12.1)	<0.001*
Exercise time during COVID-19	No data	1 (0.2)	1 (0.3)	0 (0.0)	
	Decreased	149 (28.9)	90 (23.9)	59 (42.1)	
	Did not change	204 (39.5)	166 (44.1)	38 (27.1)	
	Increased	162 (31.4)	119 (31.6)	43 (30.7)	<0.001*

*p-value significant < 0.05, and highlighted in bold. Chi square or Fischer’s exact test were used to identify significance.

### 3.4 Knowledge about nutrition and health

Knowledge about nutrition and health varied among the two generations, with a significantly higher interest in reading or following weight and nutrition related information among parents (70.7%) compared to students (45.5%, *p* < 0.001). As for the source of such information, 46.4% of parents and 32.7% of students relied on social media (*p* < 0.001), and 22.9% of parents compared to 9.6% of students obtained information from health professionals (*p* < 0.001). A notable percentage of parents (19.3%) also turned to mass media as a source of information, in contrast to 8.0% of students (*p* < 0.001). During the pandemic, there was a significant increase in the interest in nutrition and health knowledge among 36.4% of the participants, more among parents than students, respectively (46.4% vs. 32.7%, *p* = 0.001) (Table 4).

**Table 4:** Knowledge about nutrition and health in students and parents.

Knowledge on nutrition and health	Level	Total (*N* = 516)	Students (*N* = 376)	Parents (*N* = 140)	*p*-value
		*N* (%)	*N* (%)	*N* (%)	
Reading or following about weight, diet, and nutrition	No	246 (47.7)	205 (54.5)	41 (29.3)	
	Yes	270 (52.3)	171 (45.5)	99 (70.71)	<0.001*
Source of information	Health professional	68 (13.2)	36 (9.6)	32 (22.9)	<0.001*
	Social media	188 (36.4)	123 (32.7)	65 (46.4)	<0.001*
	Friends & family	28 (5.4)	17 (4.5)	11 (7.9)	<0.001*
	Mass media	57 (11.0)	30 (8.0)	27 (19.3)	<0.001*
Interest of health and nutrition effected by COVID-19	Not affected	263 (51.0)	211 (56.1)	52 (37.1)	
	Decreased	65 (12.6)	42 (11.2)	23 (16.4)	
	Increased	188 (36.4)	123 (32.7)	65 (46.4)	0.001^*^

*p-value significant (*p* < 0.05), and highlighted in bold; test used: Chi square test.

## 4 Discussion

The COVID-19 pandemic has precipitated varying eating and lifestyle shifts across global populations, with weight fluctuations, changes in eating habits, altered sleep patterns, and variable levels of physical activity reported in various populations. While some positive changes have been observed, the majority of changes signify a concerning trend towards less healthy lifestyles. These fluctuations in eating and lifestyle behaviours including physical activity and sleep patterns present potential risks especially for the younger demographic, who are often more susceptible to adopting unhealthy dietary practices. To our knowledge, this is the first Saudi study investigating eating habits, physical activity, and sleep patterns of high school students and their parents before and during COVID-19. It demonstrated the differential impacts of the COVID-19 pandemic on the eating habits and lifestyle behaviours of high school students and their parents. This highlights the need for age-specific public health strategies and educational programs to promote healthier habits and lifestyle choices, particularly for youth and vulnerable populations.

### 4.1 Eating habits among students and parents before and during COVID-19

Before COVID-19, high school students showed a marked predilection for fast food/dining out and carbonated drinks, compared to their parents. This is consistent with previous research showing that 25–80% of the Saudi teenagers reported regular consumption of fast food (8). Another Saudi study found that among Saudi women, beef or chicken burgers (70.4%) were the most popular fast-food item, followed by pizza (32.7%) and French fries (29.5%) (19). The generational effect was further demonstrated in a cross-sectional study conducted in Iran, indicating that young people consumed more fast food than their parents (20). Additionally, the high consumption of soft and carbonated drinks among Saudi youth was previously reported (9), and such beverages are often consumed within fast food meals. The idea that fast food is appealing, easy to make, and high in calories and energy is just three of the many reasons why it is popular among school-aged children (9, 21). Furthermore, fast food allows students to spend time with their friends, gives them a taste of autonomy, and gives them a sense of belonging to contemporary society and Western culture (21). Parents’ lack of interest in or knowledge of teenage food preferences, combined with their lack of time and/or money to provide traditional meals, can also play a role in shaping these preferences (21).

Regarding the effect of COVID-19 pandemic on eating habits, we observed differential shifts in eating habits between the two generations. By focusing on students’ group, one-third of them cultivated interest in health and nutrition and half of them experienced a shift in their food preference. This frequent shift to homemade food may be explained by two factors, including the lockdown context, imposing longer home stays, and the growing interest in diet. By comparison, parents exhibited a greater shift towards homemade food, which was consistent with their greater interest in health and nutrition. On the other hand, a minority of two groups reported increase in the frequency of meals or snack, with no remarkable generational effect. This suggests that COVID-19 crisis on eating habits was more likely to be qualitative than quantitative.

The review of international studies exploring the impact of COVID-19 on eating habits provides interesting observations. In the United States, the transition from before to after COVID-19 period was accompanied with several changes in the eating habits of adolescents. Specifically, there was an increase in the daily consumption of soda (from 14 to 22%) and fast food (from 7 to 10%). However, the daily intake of caffeine, fruits, vegetables, and sweets saw minimal changes (22). These shifts towards less healthy food options could have potential health implications for adolescents in the long term. Hence, the observed preference for fast food and carbonated drinks among high school students, despite parental inclinations towards healthier, home-cooked meals, seems to underscore a broader, potentially universal trend of intergenerational differences in dietary choices.

On the other hand, the closure of schools and businesses during lockdown, the extra time spent at home by students and parents, and the higher costs incurred by families as a result of the outbreak may all contribute to the improvement in the eating habits. Data from Italy showed that teenage diets had improved during COVID-19 lockdown, with fewer calories coming from fried foods and more from fruits and vegetables (23). Another survey of 2,706 Riyadh residents conducted before and after the lockdown revealed significant dietary changes, including an increase in the consumption of homemade meals, among Saudi adults. However, both the quality and quantity of the food consumed were altered (24). This variable impact of COVID-19 across the studies can be explained by the varying cultural and societal norms of the respective populations, both those related to culinary traditions and levels of nutritional awareness.

By further exploring the intergenerational effect, this shift in food preferences can be traced not only to cultural and societal factors but also to physiological changes and evolving lifestyle choices across different age groups. Interestingly, age-related differences in caloric intake and lifestyle choices can be influential factors in shaping these eating patterns. As people generally consume fewer calories with advancing age (25), this could be a significant determinant of their food choices. This notion is supported by the increased awareness about the negative aspect of junk food observed among adults (26), which may drive a preference for home-cooked meals over fast food.

### 4.2 Physical activity among students and parents during COVID-19

While the COVID-19 period was associated with decreased physical activity in a high percentage of the participants (more among parents than students), a significant proportion from both groups experienced an increase in exercise time during this period. This may be due to the increased active engagement in exercise in reaction to the imposed mobility restriction by lockdown. However, our questionnaire did not specify the timing of this shift, which could lead to variations in reported exercise time across different phases of the pandemic, with participants potentially highlighting only the most notable changes. A longitudinal study from Spain showed a 40% decrease in overall physical activity time among the study participants during lockdown (27). Another prospective study from Australia assessed the impact of COVID-19 on the physical activity of “10,000 Steps” program members and found a temporary decrease in daily steps following the first COVID-19 case and the start of lockdown. The reduction was consistent across all age groups and genders. However, steps quickly increased after restrictions were eased (28). Consistently, some studies showed increased use of parks, trails, and recreational activities among certain population groups at the start of the COVID-19 pandemic (29). However, most international research consistently shows significant decreases in mobility, walking, and physical activity, while revealing a rise in sedentary behaviour (30). This divergence in observations could be attributed to the multidimensional impact of lockdown measures on individuals’ physical activity levels. On one hand, the lockdown could stimulate individuals to participate in sports activities, driven by an increased availability of time and the need to alleviate feelings of boredom, loneliness, and anxiety through stimulating and stress-relieving activities. Conversely, the enforcement of lockdown measures necessitated the closure of fitness facilities and public parks, thereby reducing the opportunities for individuals to engage in organized physical fitness activities.

By focusing on youth, a systematic review of 51 international articles indicated a global decline in physical activity during the COVID-19 pandemic. Critical findings include a lower percentage of adolescents meeting the World Health Organization (WHO) physical activity guidelines, along with gender differences favoring boys in exercise levels. Furthermore, vigorous physical activity was associated with reduced anxiety during stressful periods, as noted in the U.S. Furthermore, the levels of physical activity have significant implications for mental health during the pandemic period (31). Another prospective study observed an overall decline in physical activity among adolescents during the COVID-19 lockdown. However, such impact was significantly more remarkable among younger adolescents (aged 14–16 years) compared to their older counterparts (16–18 years). Furthermore, authors identified parents’ education and before COVID-19 engagement to be protective factors against physical activity decline (32).

To understand these effects, Teare and Taks used the Social Ecology Theory to explain the shift in recreational activities among the youth during the COVID-19 lockdown. They identified several intrapersonal and interindividual factors that shaped the dynamics of exercise preferences and behaviours during the pandemic. This was combined with political, institutional factors that influenced access to sports facilities, which had financial and community implications and played an additional role in shaping the youths’ activities and preferences (33). Besides, the impact of COVID-19 on physical activity among youth was associated with other detrimental behaviours, particularly increased sedentary recreational activities such as gaming (34).

From a public health perspective, these observations highlight the importance of physical activity for youth’s overall health, the need for adaptable exercise options, the significant role of parents and home environments, and the potential of technology to improve accessibility.

### 4.3 Poor sleep quality in Saudi students

Findings from this study showed a significant disparity in sleeping patterns between students and adults (*p* < 0.05). This denotes the susceptibility of the younger populations to delayed sleep phase syndrome, characterized by a shift in sleep schedule towards later hours, which can be further exacerbated by factors such as increased screen time (35), academic pressure (36), and disrupted psychosocial well-being (37). Local studies conducted pre-pandemic in Jeddah and Riyadh observed prevalent sleep disturbances among Saudi adolescents, with 65% reporting issues and an additional 37% experiencing excessive daytime sleepiness, while around one-third of adult participants reported sleeping for less than 7 h per night (38, 39). This indicates a rampant suboptimal sleep quality among both adolescent and adult populations in Saudi Arabia, which is probably due to the local culture and lifestyle. However, it is worth noting that there appears to be a higher prevalence of sleep deficiency among adolescents like one observed in the present study. A study from Turkey showed that 55.5% of school-aged children experienced sleep disturbances during the COVID-19 pandemic, with younger children being more susceptible. Several socioeconomic and psychological factors have been linked to these disturbances, underlining the importance of education and social and mental health support for families to mitigate sleep disturbances in children during periods of crisis (40). These findings emphasize the importance of mental health support, healthy lifestyle education, and balanced screen use to address sleep disturbances, which can have significant implications for the physical and mental health, academic achievements, and overall well-being of school-aged children.

### 4.4 Limitations

This study has several limitations that should be acknowledged. First, the cross-sectional design used to collect before COVID-19 data may be subject to recall bias, potentially affecting the reliability of participant responses. Second, the self-reported questionnaire may introduce subjectivity and potential inconsistency. Third, our research design did not strictly adhere to a 1:1 ratio of students to parents, which may reduce the statistical power of the comparative analysis. We also did not perform a paired analysis, which would compare each student with his/her parent, thereby offering a more precise comparison by eliminating the potential variances that may be attributed to individual household parameters. Moreover, the absence of information about non-participants and their characteristics in comparison to participants, which could enhance the generalizability of the findings.

Further limitations relevant to the context that need to be highlighted is that the data was collected three years ago during the height of the COVID-19 pandemic. While this provides valuable insights into eating habits during an unprecedented time, the long-term effects and current trends may have evolved since then. Indeed, there is a potential for significant changes in eating habits and food availability post-pandemic, which compromises the current relevance of the findings to the target population of young individuals aged 15–18 years old. The perspective of this age group, particularly their adaptive behaviours and new eating patterns post-pandemic, may not be fully captured in the study. Ongoing or future studies would be necessary to capture these changes and provide more current insights.

Lastly, it is essential to emphasize the challenges in capturing real-time data during the pandemic. Despite these limitations, insights gained from this study can still contribute to the broader understanding of pandemic impacts on eating habits and, more broadly, lifestyle behaviours including physical activity and sleep patterns among the adolescent group. These limitations provide important context for interpreting the findings of this study.

## 5 Conclusion

There are considerable differences in eating habits, physical activity, and sleep patterns among high school students and their parents, before and during the COVID-19 pandemic. While parents demonstrated a significant increase in health and nutrition awareness, students showed higher tendencies towards unhealthy behaviours. These insights highlight the differential impact of the pandemic across the population age groups, underscoring the need for generation-specific health promotion interventions considering the unique challenges and behaviours. Future research, with a more rigorous design, is warranted to further explore these intergenerational differences and develop effective strategies to mitigate unhealthy lifestyle patterns and their dynamic shifts during crisis periods.

## Data availability statement

The datasets presented in this study can be found in online repositories. The names of the repository/repositories and accession number(s) can be found in the article/supplementary material.

## Ethics statement

The studies involving humans were approved by Research Ethics Committee of the Faculty of Applied Medical Sciences at King Abdulaziz University, under the reference number FAMS-EC2021-15. The studies were conducted in accordance with the local legislation and institutional requirements. Written informed consent for participation in this study was provided by the participants’ legal guardians/next of kin.

## Author contributions

SA: Conceptualization, Formal analysis, Investigation, Methodology, Supervision, Validation, Writing – original draft, Writing – review & editing.
